# Effect of age on response to anti-VEGF agents in patients with center involving diabetic macular edema in a tertiary hospital

**DOI:** 10.1186/s40942-022-00434-9

**Published:** 2022-12-13

**Authors:** Hanan A. Alshalan, J. Fernando Arevalo, Saleh I. Alomary, Husam I. Ardah, Mohammd A. Hazzazi

**Affiliations:** 1 Vitreoretinal Division, King Khaled Eye Specialist Hospital, Riyadh, Saudi Arabia; 2 Wilmer Eye Institute, Johns Hopkins University, Baltimore, MD USA; 3 Department of Family Medicine, King Abdulaziz Medical City, Riyadh, Saudi Arabia; 4 King Abdullah International Medical Research Center (KAIMRC), Riyadh, Saudi Arabia; 5 Department of Ophthalmology, King Abdulaziz Medical City, Riyadh, Saudi Arabia; 6 King Saud Bin Abdulaziz University for Health Sciences, Riyadh, Saudi Arabia

## Abstract

**Purpose:**

The aim of the current study is to evaluate the effect of age as an independent factor for the response to two anti-VEGF agents, intravitreal ranibizumab and intravitreal aflibercept, among patients presented with central-involving DME in one tertiary care center in Riyadh, Saudi Arabia.

**Methods:**

Retrospective cohort study.

**Results:**

A total of 210 eyes of 121 patients were included in the study. The mean age was 61.2 ± 11.40 years, 50.4% were males. On characterizing groups based on age, the group of patients 60 years or younger are 48 patients (mean age 51.5 ± 9.92) and 52.1% of them are females. On the other hand, the group of patients older than 60 years are 73 patients (mean age 67.6 ± 6.85) and 52.1% of them are males. The two anti-VEGF agents used were aflibercept (88.1%) and ranibizumab (11.9%). The mean BCVA using ETDRS letter score improved after treatment (5.55238095 ± 15.9538695) and the mean change in CST decreased after treatment (− 106.91 ± 117.385 μm). Regarding age, we found that there is no significant difference in mean improvement of BCVA in patients according to their age (*p* = 0.5429), patients younger than 60 years old gained 5.64 ETDRS letter score and those older than 60 years old gained 5.49 improvement. Similarly, mean improvement in CST was different between patients younger than 60 years old (− 125.1 μm) and those who were older than 60 years old (− 94 μm) with a trend favoring younger patients but this difference was not statistically significant (*p* = 0.08).

**Conclusion:**

Age is a clinically significant factor affecting the outcome of anti-VEGF injections. Patients’ CST had a difference of > 30 μm on average between the two age groups favoring younger patients. However, it was not statistically significant, maybe a bigger sample size is needed to prove statistical significance.

## Introduction

Diabetic macular edema (DME) is the most common cause of central vision impairment in patients with diabetic retinopathy and can occur at any stage of the disease [1]. Although the exact pathogenesis of DME is not yet understood, there is increased vascular permeability of the retinal capillaries, as a result of breakdown of the blood retinal barrier that leads to accumulation of extracellular fluid in the macula [2]. Furthermore, prolonged hyperglycemia causes reduction in inner retinal oxygen tension, venous dilation and upregulation of the expression of vascular endothelial growth factor (VEGF), which altogether increase microvascular leakage [2].

Intravitreal ranibizumab (Lucentis, Genentech, South San Francisco, California, USA) was the first anti-VEGF agent approved by the US Food and Drug Administration (FDA) in 2006 for the treatment of diabetic macular edema (DME) [3]. Later, in 2011, Intravitreal aflibercept (EYLEA, Regeneron, Tarrytown, NY) was approved by FDA following large double-masked, randomized phase III clinical trials VISTA-DME and VIVID-DME [4, 5].

While treatment with anti-VEGF agents is often very effective, some patients fail to respond, presenting refractory or persistent DME, which often require other forms of medical management, including alternative anti-VEGF therapies or intravitreal steroids [6].

A few studies have evaluated some factors that may influence the response to anti-VEGF agents in DME patients, whereas the results are inconsistent among these studies and inconclusive [7–9]. Most studies suggested that poor baseline visual acuity (≤ 20/125) and higher baseline thickness on optical coherence tomography predicts a poor outcome [7, 8]. One study published on February 2020 revealed several optical coherence tomography characteristics such as the presence of serous retinal detachment, hyperreflective retinal spots, the disruption of external limiting membrane and ellipsoid zone, the disorganization of inner retinal layers and continued increase in macular thickness, as predictors of poor response to treatment [10].

In addition, one study found that age might be correlated with outcome to anti-VEGF agents in DME patients but had a small sample size and potential selection bias [11]. This result was consistent with some results of previous studies which found that younger age tend to have a higher visual gain in DME [7].

The aim of the current study was to evaluate the effect of age as an independent factor for the response to two anti-VEGF agents, intravitreal ranibizumab and intravitreal aflibercept, among patients presented with central-involving DME in one tertiary care center in Riyadh, Saudi Arabia.

## Methods

This retrospective cohort study was conducted at King Abdulaziz Medical City (KAMC), Riyadh, Saudi Arabia. Ethical approval for this study was obtained from Institutional Review Board at King Abdullah International Medical Research Center (KAIMRC). The study adhered to the tenets of Declaration of Helsinki.

Electronic medical records were queried for patients with type 1 or type 2 diabetes mellitus, 20 years of age or older, who presented to the retina department with central-involving diabetic macular edema (DME) that required treatment with anti-vascular endothelial growth factor (anti-VEGF) agent, between 2016 and 2020. Patients with concomitant retinal disease that can affect the macula independently other than diabetic retinopathy were excluded.

A standardized protocol using records of patients with a confirmed diagnosis of type 1 or 2 diabetes were utilized to extract information on age, gender, insulin dependency, and glycated hemoglobin level for each case.

Data on optical coherence tomography imaging (OCT; Spectralis OCT, Heidelberg Engineering, Inc, Heidelberg, Germany) features of DME, color fundus photography (Optos PLC, Dunfermline, UK), and best corrected visual acuity (BCVA) corresponding to the pre, and post treatment visits were collected. Patients who received anti-VEGF therapy but were lost follow up in which we cannot evaluate the response were excluded.

OCT features including central subfield thickness (CST), disorganization of retinal inner layers (DRIL), hyperreflective foci (HRF), and subretinal fluid (SRF) were compared before and after intervention. Visual acuity was analyzed using standardized Early Treatment of Diabetic Retinopathy Study (ETDRS) letter score.

Two anti-VEGF agents were used; intravitreal ranibizumab and intravitreal aflibercept. Regarding age, we collected age of each patient, patients with missing age were excluded.

### Statistical analysis

Patients were divided into two groups according to age, patients at 60 years old or younger and patients older than 60 years old. Means and proportions of the study participants were calculated to characterize the study participants, overall and in groups. The two groups (≤ 60 years, > 60 years) were compared using Chi square or fisher exact test for categorical factors and *t*-test or Kruskal Wallis Test for continuous variables as appropriate. Then, eyes, the study observations, were also divided into two groups based on patients’ age. Again, means and proportions of the observed eyes indicators were calculated to characterize the treated eyes, overall and in groups. The two groups (≤ 60 years, > 60 years) were compared using simple linear mixed models to account for the within patient correlations as appropriate. simple linear mixed models were also used to study the effect of set of factors on the improvement of BCVA and CST. Level of significance was declared at α = 0.05. Statistical analysis was conducted using SAS 9.4 (SAS Institute Inc., Cary, NC, USA).

## Results

A total of 210 eyes of 121 patients were included in the current study. The demographics of the study group are listed in Table 1. The mean age was 61.2 ± 11.40 years, and 50.4% were males.

**Table 1:** Variables (based on patients)

	≤ 60 years = 48	> 60 years = 73	Total = 121	*p*-value
Age				
Mean (SD)	51.5 (9.92)	67.6 (6.85)	61.2 (11.40)	< .0001^a^
Gender				
M	23 (47.9)	38 (52.1)	61 (50.4)	0.6560
F	25 (52.1)	35 (47.9)	60 (49.6)	0.6560
HgA1C				
Mean (SD)	9.4 (1.47)	8.9 (1.67)	9.1 (1.61)	0.1016
SystemicRx				
Insulin	45 (93.8)	72 (98.6)	117 (96.7)	0.2996
OHA	3 (6.3)	1 (1.4)	4 (3.3)	0.2996
HTN				
Present	35 (72.9)	65 (89.0)	100 (82.6)	0.0220
DLP				
Present	40 (83.3)	67 (91.8)	107 (88.4)	0.1553
IHD				
Present	4 (8.3)	17 (23.3)	21 (17.4)	0.0336

^a^Age, age of study participants; Gender M, males; F, females; HgA1C, hemoglobin A1C; Insulin, patients on insulin; OHA, patients on oral hypoglycemic agents; HTN, hypertension; DLP, dyslipidemia; IHD, ischemic heart disease

On characterizing groups based on age, the group of patients 60 years old or younger included 48 patients (mean age 51.5 ± 9.92) and 52.1% of them are females. On the other hand, the group of patients older than 60 years included 73 patients (mean age 67.6 ± 6.85) and 52.1% of them are males. Hemoglobin A1C was found to be on average higher in the younger group. Furthermore, 93.8% of patients 60 years or younger were insulin dependent, in comparison to 98.6% in older group (*p* = 0.29).

The most prevalent comorbidities found in the study group were hypertension, dyslipidemia, and ischemic heart disease. On group analysis, 72.9% of patients 60 years or younger were hypertensive compared to 89% of patients older than 60 years (*p* = 0.022). Additionally, 83.3% of patients 60 years or younger had dyslipidemia, while 91.8% in the older group did (*p* = 0.155). Finally, 8.3% in the younger group had ischemic heart disease compared to 23.3% in the older group (*p* = 0.033) (Table 1).

Majority of eyes in this study were found to have moderate non-proliferative diabetic retinopathy (48.1%) on fundus examination. Based on age groups, proliferative diabetic retinopathy was detected in 26.7% of young group and 17.7% of older group. Adjunctive focal laser treatment was used in 24.8% of eyes in total with no significant difference between the two groups (*p* = 0.4086).

Injection to OCT interval was on average 10.1 ± 6.75 weeks in the young group while it was 13.4 ± 14.6 weeks in the older group (*p* = 0.09). The number of injections was calculated in each group and was found to be 4.7 ± 1.80 on average in the young group, and 4.2 ± 1.79 on average in the older group with no significant difference (*p* = 0.09). The two anti-VGEF agents used were aflibercept and ranibizumab (Table 2).

**Table 2:** Variables (based on eyes)

	≤ 60 years = 86	> 60 years = 124	Total = 210	*p*-value
No DR	0 (0)	4 (3.2)	4 (1.9)	0.1575
Mild NPDR	14 (16.3)	14 (11.3)	28 (13.3)	0.1575
Moderate NPDR	33 (38.4)	68 (54.8)	101 (48.1)	0.1575
Severe NPDR	16 (18.6)	16 (12.9)	32 (15.2)	0.1575
PDR	23 (26.7)	22 (17.7)	45 (21.4)	0.1575
Focal laser	24 (27.9)	28 (22.6)	52 (24.8)	0.4086
Injection to OCT interval, mean (SD)	10.1 (6.75)	13.4 (14.60)	12.0 (12.11)	0.0998
No. of injections, mean (SD)	4.7 (1.80)	4.2 (1.79)	4.4 (1.81)	0.0986
Aflibercept	78 (90.7)	107 (86.3)	185 (88.1)	0.3321
Ranibizumab	8 (9.3)	17 (13.7)	25 (11.9)	0.3321

No DR, no diabetic retinopathy; Mild NPDR, mild nonproliferative diabetic retinopathy; Moderate NPDR, moderate nonproliferative diabetic retinopathy; Severe NPDR, severe nonproliferative diabetic retinopathy; PDR, proliferative diabetic retinopathy; Focal laser, performed; Injection to OCT interval, in weeks; No. of injections, number of injections received; Aflibercept, patients received Aflipercept; Ranibizumab, patients received Ranibizumab

**Fig. 1:**
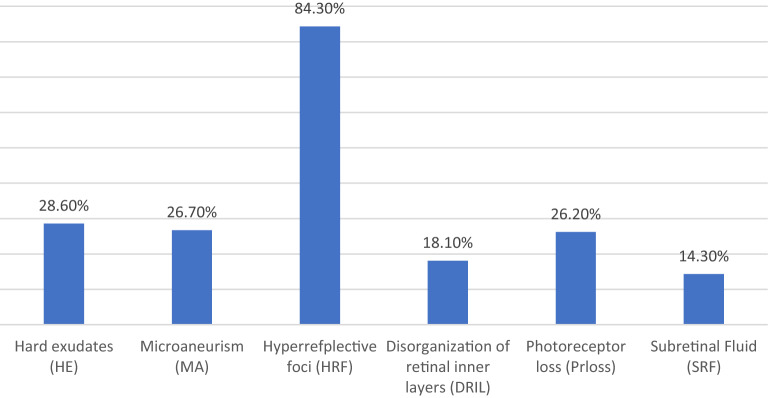
Diabetic macular edema characteristics

The characteristics of diabetic macular edema in the 121 patients included in this study are listed in Fig. 1. On Fundus examination, hard exudates and microaneurysms were seen in 28.6% and 26.7% respectively. The most common noticeable feature in OCT was hyper-reflective foci present in 84.3% of eyes. Subretinal fluid was the least common feature present, seen in 14.3% only.

Best corrected visual acuity was analyzed using standardized Early Treatment of Diabetic Retinopathy Study (ETDRS) letter score. The mean BCVA improved after treatment (5.5 ± 15.9). Considering age, we found that there is no significant difference in mean improvement of BCVA in patients according to their age (*p* = 0.5429), patients younger than 60 years old had 5.64 ETDRS letter score and those older than 60 years old had 5.49 ETDRS letter score. Similarly, the mean change in CST decreased after treatment (− 106.9 ± 117.3 μm) but the difference between the two groups was not statistically significant (*p* = 0.08).

When analyzing CST difference based on gender, the mean reduction was − 101.7 μm in male group and − 112.1 μm in female group, and the difference between the two groups was not statistically significant (*p* = 0.5542). When analyzing BCVA difference based on gender, the mean improvement was 4.04 in male group and 7.07 in female group, and the difference between the two groups was not statically significant (*p* = 0.1910). The presence of DRIL was significantly associated with more reduction in CST (− 156.4 ± 159.5 μm, *p* < 0.031) but without significant increase in ETRDS score (7.1 ± 19.4, *p* = 0.2941). Similarly, in eyes with SRF and photoreceptor loss, the reduction in CST was significantly better (− 166.1 ± 154.1 μm, *p* < 0.0031) and (− 144.6 ± 133.8 μm, *p* = 0.0037) respectively. As for ETDRS letter score, the presence of SRF was significantly associated with increase in ETDRS letter score after treatment (12.4 ± 15.4, *p* = 0.0089). On the other hand, the presence of photoreceptor loss yields no significant improvement (5.3 ± 17.9, *p* = 0.9230). The presence of hyper-reflective foci did not yield a significant difference in CST nor ETDRS score (*p* = 0.4170 and 0.4426 respectively). The performance of focal laser in 52 eyes did not affect the ETDRS (*p* = 0.4281) or CST (*p* = 0.7805) outcome.

Lastly, presence of other comorbidities between the two groups did not yield a statistically significant difference. For example, the analysis of the difference in CST and ETDRS score between hypertensive and non-hypertensive patients in the two groups was not significant (*p* = 0.7434 and 0.5123) respectively. Similar results were noted for dyslipidemia and ischemic heart disease, which are the three most prevalent comorbidities in this study (Table 3).

**Table 3:** Factors affecting mean improvement of central subfield thickness and best corrected visual acuity after the treatment

Factor	Post–pre CST^a^		Post–pre BCVA^b^	
	Mean	*p*-value	Mean	*p*-value
Gender				
Male	− 101.75	0.5542	4.04	0.1910
Female	− 112.08		7.07	
Age				
≤ 60 years	− 125.1	0.0876	5.64	0.5429
> 60 years	− 94		5.49	
DRIL				
Absent	− 95.98	0.0031	5.20	0.2941
Present	− 156.42		7.13	
HRF				
Absent	− 89.70	0.4170	3.64	0.4426
Present	− 110.12		5.91	
SRF				
Absent	− 97.04	0.0053	4.41	0.0089
Present	− 166.13		12.43	
Pr loss				
Absent	− 93.96	0.0037	5.59	0.9230
Present	− 144.65		5.35	
Focal Laser				
Absent	− 108.44	0.7805	6.08	0.4281
Present	− 102.27		3.94	
HTN				
Absent	− 95.7143	0.7434	3.5429	0.5123
Present	− 109.2		5.9543	
DLP				
Absent	− 132.2	0.3530	5.9231	0.9202
Present	− 103.3		5.5000	
IHD				
Absent	− 112.7	0.1848	6.5549	0.0825
Present	− 79.8108		0.8649	

^a^BCVA, best-corrected visual acuity

^b^CST, central subfield thickness; < 60 years, patients who are 60 years or younger; > 60 years, patients who are older than 60 years; DRIL, disorganization of retinal layers; HRF, hyperreflective foci; SRF, subretinal fluid; Pr loss, photoreceptors loss; focal laser, focal laser performance, HTN, hypertension; DLP, dyslipidemia; IHD, ischemic heart disease

On analysis based on injection type, ranibizumab was used in 90.7% of patients younger than 60 years and in 86.3% of patients older than 60 years with no significant difference (*p* = 0.332). The mean increase in ETDRS letter score after ranibizumab was (0.64 ± 10.3) while after aflibercept it was (6.2 ± 16.4), and this difference was found to be statically significant (*p* = 0.0245). The mean reduction in CST after ranibizumab was (− 67.0400 ± 77.8832) while for aflibercept was (− 112.3 ± 120.9) and this also was statically significant (*p* = 0.0155).

When we compared the impact of each injection on improvement in BCVA and CST according to age, we found that the mean improvement of BCVA and CST in case of ranibizumab was significantly higher in patients younger than 60 (− 228.13 and 19.13 respectively) than in patients older than 60 years old (− 100.29 and 4.18 respectively) (*p* = 0.001) while in case of aflibercept, the difference was not significant (*p* = 0.356) however improvement of BCVA in patients older than 60 years old was higher (5.7 vs. 4.26) and lower in case of CST (− 91.4 vs. − 117.21) than patients younger than 60 years old (Table 4).

**Table 4:** The improvement of BCVA and CST in patients using Ranibizumab and Aflibercept according to age

	Age			
	< 60		> 60	
	Post–pre-BCVA*	Post–pre-CST*	Post–pre-BCVA	Post–pre-CST
	Mean	Mean	Mean	Mean
Injection type				
Ranibizumab	19.13	− 228.13	4.18	− 100.29
Aflibercept	4.26	− 117.21	5.70	− 91.40

*BCVA, best-corrected visual acuity, CST, central subfield thickness

## Discussion

In this study, a total of 210 eyes of 121 patients were included. The mean BCVA using ETDRS letter score improved after treatment (5.55238095 ± 15.9538695) and the mean change in CST decreased after treatment (− 106.91 ± 117.385 μm) in all patients. Regarding age, we found that there is no significant difference in mean improvement of BCVA in patients according to their age (*p* = 0.5429), patients younger than 60 years old had 5.64 ETDRS letter score and those older than 60 years old had 5.49 improvement. Similarly, mean improvement in CST was not significantly different between patients younger than 60 years old (− 125.1 μm) and those who were older than 60 years old (− 94 μm) with a *p*-value of 0.08.

Although, many previous studies had proven the benefits of anti-VEGF therapy in the management of central involving DME in diabetes patients [12–14], there is a subgroup of patients who had persistent DME after the initiation of the medication [15]. In a previous study, a secondary analysis of Protocol T had been conducted showing that after six monthly intravitreal injection of anti-VEGF, persistent macular thickening was found in 65.6%, 41.5% and 31.6% of eyes treated with bevacizumab, ranibizumab, and aflibercept, respectively [16]. The clinical challenge in finding factors that could predict the individual response to anti-VEGF treatment remains where the importance of finding these factors represented by physicians’ ability to counsel patients and manage their expectations. The aim of the current study was to evaluate the effect of age as an independent factor for the response to two anti-VEGF agents, intravitreal ranibizumab and intravitreal aflibercept, among patients presented with central-involving DME in one tertiary care center in Riyadh, Saudi Arabia.

The baseline characteristic of the patients showed that the mean age of the patients was 61.2 years where 59% of them were older than 60 years old. These are similar to other reports that reported that most of patients with central involving DME were older than 60 years old [7, 9, 17]. The administration of aflibercept was done in 88.1% of cases compared with 11.9% of ranibizumab in our series. In general, after treatment, the mean BCVA improved was (5.5 ± 15.9) and mean change in CST decreased after treatment (− 106.9 ± 117.3 μm). No significant difference was found in improvement of CST and VA according to age or gender, presence of HFR or having previous focal laser. The insignificant impact of gender on improvement of VA and CST was reported in previous studies including a study by Bressler et al. [7], and a study by Chen et al. [11] who reported that gender is not associated with response to treatment. However, a study by Sophie et al. [9] found that better results were found after the use of ranibizumab in male patients (OR, 1.85, *p* = 0.005) as well as results from the study by Channa et al. [8] who showed better CST results after treatment in cases who initially had DRIL, SRF and photoreceptor loss at baseline characteristics. Considering the impact of age on the CST and VA, we found that older patients (older than 60 years) had poorer results after treatment with anti-VEGF with lower CST improvement but that failed to show statistical significance. These results were in consistent with the results by Chen et al. [11] who found that the treatment response was significantly affected by age where better outcomes were reported in younger patients. Another study conducted by Bressler et al. [7] showed that better treatment outcomes were found in patients younger than 60 years old. However, it is unknown why better outcomes are related to younger patients in any of these studies.

In addition, we found significant difference between CST and VA results for each anti-VEGF agents utilized where better results were significantly reported in patients treated with aflibercept than in patients treated with ranibizumab. In a study by Demircan et al. [18] the authors showed that there was significant difference considering CMT reduction between patients treated with aflibercept (188.6 ± 120.5 μm reduction after treatment) as compared to patients treated with ranibizumab (60.3 ± 117.1 μm reduction after treatment). A study conducted by Bahrami et al. similarly reported the beneficial results of aflibercept on both visual improvement in addition to morphologic improvement in patients with DME who had poor response to previous bevacizumab injections [19]. Moreover, Wood et al. [20] reported only morphologic improvement with administration of aflibercept in patients with poor response to ranibizumab and/or bevacizumab injections. Rahimy et al. [21] also found only a morphologic response to aflibercept injections after previous bevacizumab/or ranibizumab therapy, and they showed that this result could be explained by irreversible functional damage caused by long-standing DME. However, when comparing these results according to age, we found that the mean improvement of VA and CST in case of ranibizumab was significantly higher in patients younger than 60 (− 228.13 and 19.13 respectively) than patients older than 60 years old (− 100.29 and 4.18 respectively) (*p* = 0.001) while in case of aflibercept, the difference was not significant (*p* = 0.356) however improvement of VA in patients older than 60 years old was higher (5.7 vs. 4.26) with lower numbers in case of CST (− 91.4 vs. − 117.21) than patients younger than 60 years old. These results demonstrated that aflibercept treatment showed favorable vision outcomes however ranibizumab is better in patients younger than 60 years old, however the number of patients is small in Ranibizumab group.

This study has some limitations including its retrospective nature and therefore some data were missed from the electronic medical records and could not be retrieved such as the duration of diabetes mellitus. Another factor is the relatively small number of patients especially in the ranibizumab group. However, our results constitute evidence derived from a real-world setting and provide additional information, complementing the findings of clinical trials, to help guide physicians in their routine clinical practice. Another limitation was the possibility of selection bias, especially when comparing the treatment outcomes between different anti-VEGF agents.

In conclusion, we found that the intervention of anti-VEGF injections was associated with better outcomes of vision improvement especially in case of using aflibercept. Age is a significant clinical factor affecting the outcomes of anti-VEGF intervention in clinical practice, however this failed to reach statistical significance in our study. The results of the study showed that aflibercept could be used for all age groups of patients with no significant difference while the use of ranibizumab maybe better restricted to younger patients.

## Data Availability

All data generated during this study are included in this published article.
